# Supplementary material for: The Systems Biology Research Tool: evolvable open-source software
Source: BMC Syst Biol. 2008 Jun 29;2:55. doi: 10.1186/1752-0509-2-55 (PMC2446383; doi:10.1186/1752-0509-2-55)
Supplement: Additional file 1 — SBRT Archive. An archive of the current version of the Systems Biology Research Tool. [file 1752-0509-2-55-S1.zip › sbrt-1.4.0/doc/developers_guide/api/sbrt/kernel/fba/proc/FluxPlasticityProcess.Results.html]

FluxPlasticityProcess.Results


|  |  |  |
| --- | --- | --- |
| **Overview** \| **Package** \| **Class** \| **Use** \| **Tree** \| **Deprecated** \| **Index** \| **Help** | |  |
| **PREV CLASS**   **NEXT CLASS** | **FRAMES**    **NO FRAMES**     **All Classes** |  |
| SUMMARY: NESTED \| FIELD \| CONSTR \| METHOD | DETAIL: FIELD \| CONSTR \| METHOD |  |


---


## sbrt.kernel.fba.proc Class FluxPlasticityProcess.Results

```
java.lang.Object
  sbrt.kernel.fba.proc.FluxPlasticityProcess.Results
```

**Enclosing class:**: FluxPlasticityProcess

---

``` public static class FluxPlasticityProcess.Results extends java.lang.Object ```

This class is used to represent the results of a flux plasticity
experiment.

**Author:**
:   This class was written and documented by Jeremiah Wright while in
    the Wagner lab.

---

| **Method Summary** | |
| --- | --- |
| `int` | `alwaysActive()`             Returns the number of fluxes that are always active. |
| `UnmodifiableSet<java.lang.String>` | `getAlwaysActive()`             Returns the set of fluxes that are always active. |
| `UnmodifiableSet<java.lang.String>` | `getNeverActive()`             Returns the set of fluxes that are never active. |
| `UnmodifiableSet<java.lang.String>` | `getPotActive()`             Returns the set of fluxes that are potentially active. |
| `int` | `neverActive()`             Returns the number of fluxes that are never active. |
| `int` | `plastic()`             Returns the number of plastic fluxes. |
| `int` | `potentiallyActive()`             Returns the number of fluxes that are potentially active. |
| `int` | `rigid()`             Returns the number of rigid fluxes. |

**Methods inherited from class java.lang.Object**

`clone, equals, finalize, getClass, hashCode, notify, notifyAll, toString, wait, wait, wait`

**Method Detail**

### plastic

```
public int plastic()
```

:   Returns the number of plastic fluxes.

    :   **Returns:**: the number of plastic fluxes.

---


### rigid

```
public int rigid()
```

:   Returns the number of rigid fluxes.

    :   **Returns:**: the number of rigid fluxes.

---


### alwaysActive

```
public int alwaysActive()
```

:   Returns the number of fluxes that are always active. Always-active
    fluxes are those whose lower bound is greater than zero.

    :   **Returns:**: the number of fluxes that are always active.

---


### potentiallyActive

```
public int potentiallyActive()
```

:   Returns the number of fluxes that are potentially active.
    Potentially-active fluxes are those whose lower bound is zero and
    whose upper bound is greater than zero.

    :   **Returns:**: the number of fluxes that are potentially active.

---


### neverActive

```
public int neverActive()
```

:   Returns the number of fluxes that are never active. Never-active
    fluxes are those whose upper bound is zero.

    :   **Returns:**: the number of fluxes that are never active.

---


### getAlwaysActive

```
public UnmodifiableSet<java.lang.String> getAlwaysActive()
```

:   Returns the set of fluxes that are always active. Always-active
    fluxes are those whose lower bound is greater than zero.

    :   **Returns:**: the set of fluxes that are always active.

---


### getPotActive

```
public UnmodifiableSet<java.lang.String> getPotActive()
```

:   Returns the set of fluxes that are potentially active.
    Potentially-active fluxes are those whose lower bound is zero and
    whose upper bound is greater than zero.

    :   **Returns:**: the set of fluxes that are potentially active.

---


### getNeverActive

```
public UnmodifiableSet<java.lang.String> getNeverActive()
```

:   Returns the set of fluxes that are never active. Never-active
    fluxes are those whose upper bound is zero.

    :   **Returns:**: the set of fluxes that are never active.


---


|  |  |  |
| --- | --- | --- |
| **Overview** \| **Package** \| **Class** \| **Use** \| **Tree** \| **Deprecated** \| **Index** \| **Help** | |  |
| **PREV CLASS**   **NEXT CLASS** | **FRAMES**    **NO FRAMES**     **All Classes** |  |
| SUMMARY: NESTED \| FIELD \| CONSTR \| METHOD | DETAIL: FIELD \| CONSTR \| METHOD |  |


---
